# Robust automated preclinical fMRI preprocessing via a multi-stage dilated convolutional Swin Transformer affine registration

**DOI:** 10.3389/fnins.2025.1621244

**Published:** 2025-12-12

**Authors:** Sima Soltanpour, Md. Taufiq Nasseef, Rachel Utama, Arnold Chang, Dan Madularu, Praveen Kulkarni, Craig F. Ferris, Chris Joslin

**Affiliations:** 1 School of Information Technology, Carleton University, Ottawa, ON, Canada; 2 Department of Mathematics, College of Science and Humanity Studies, Prince Sattam Bin Abdulaziz University, Riyadh, Saudi Arabia; 3 Center for Translational NeuroImaging (CTNI), Northeastern University, Boston, MA, United States; 4 Tessellis Ltd., Ottawa, ON, Canada

**Keywords:** functional MRI, preprocessing pipeline, affine registration, transformers, deep learning

## Abstract

**Introduction:**

Accurate preprocessing of functional magnetic resonance imaging (fMRI) data is crucial for effective analysis in preclinical studies. Key steps such as denoising, skull-stripping, and affine registration are essential to align fMRI data with a standard atlas. However, challenges such as low resolution, variations in brain geometry, and limited dataset sizes often hinder the performance of traditional and deep learning-based methods.

**Methods:**

To address these challenges, we propose a preclinical fMRI preprocessing pipeline that integrates advanced deep learning modules, with a particular focus on a newly developed Swin Transformer-based affine registration method. The pipeline incorporates our previously established modules for 3D Generative Adversarial Network (GAN)-based denoising and Transformer-based skull stripping, followed by the proposed Multi-stage Dilated Convolutional Swin Transformer (MsDCSwinT) for affine registration. This new registration method captures both local and global spatial misalignments, ensuring accurate alignment with a standard atlas even in challenging preclinical datasets.

**Results:**

We validate the pipeline across multiple preclinical fMRI studies and demonstrate that our affine registration module achieves higher average Dice similarity coefficients compared to state-of-the-art methods.

**Discussion:**

By leveraging GANs and Transformers, our pipeline offers a robust, accurate, and fully automated solution for preclinical fMRI.

## 1 Introduction

Small animal models play a crucial role in preclinical research, aiding in the evaluation of new pharmaceutical compounds and the investigation of biological functions (Hasani et al., 2025). Rats are one of the most commonly used species in medical studies due to their compact size, rapid reproduction, genetic resemblance to humans, and their ability to model various human diseases (Szpirer, 2020). Advancements in imaging technologies enable the continuous, non-invasive examination of both anatomical structures and biological processes in small animals. Functional magnetic resonance imaging (fMRI) is a non-invasive imaging tool used to assess brain structure and function, which works with magnetic fields and radiofrequency pulses (Ren et al., 2022).

fMRI preprocessing is a critical step in neuroimaging analysis, ensuring that raw fMRI data is corrected for artifacts, aligned to a standard space, and optimized for subsequent statistical and machine learning-based analyses. A typical fMRI preprocessing pipeline includes essential steps such as denoising, motion correction, skull-stripping, and registration to an atlas. These steps are crucial for minimizing variability across scans, improving signal quality, and enabling accurate group-level comparisons (Nieto-Castanon, 2022; Di and Biswal, 2023). However, traditional preprocessing methods, including conventional optimization-based registration and CNN-based approaches, often struggle with low-resolution fMRI data, anatomical variations, and limited sample sizes, particularly in preclinical studies.

Rigid and affine registration play a key role in medical imaging and have been widely studied for years. Deep learning has demonstrated advancements in medical image registration. Some existing approaches rely on supervised learning frameworks, which require extensive annotated datasets and domain-specific expertise (Chen et al., 2021, 2022). Supervised learning approaches for image registration depend heavily on accurately labelled data, which poses a significant challenge in fMRI analysis due to the variability in manually segmented maps across different brain regions. As a result, such methods may be impractical in scenarios where ground-truth registration labels are unavailable, as is the case in this study. To address this limitation, unsupervised and weakly supervised learning strategies have gained attention as viable alternatives, reducing the reliance on precise manual annotations while still enabling effective image alignment (Mok and Chung, 2022; Ji and Yang, 2024; Golestani et al., 2025). Although these approaches offer potential advantages over supervised methods, their applicability to preclinical fMRI image registration remains unexplored and requires further research (Chen et al., 2021). Moreover, weakly supervised registration methods rely on segmentation labels as supervision, making their performance highly sensitive to segmentation variability. In fMRI datasets, where the number and shape of segmented regions can vary significantly across subjects, this dependence further degrades consistency and accuracy. Therefore, adopting an unsupervised learning strategy is more appropriate for achieving robust registration in preclinical fMRI analysis.

In many image registration systems, images are first aligned using rigid or affine transformations before applying non-rigid or deformable methods (De Vos et al., 2019). This step helps correct large-scale misalignments between images (Mok and Chung, 2022). Recent learning-based methods for deformable image registration rely heavily on accurate affine alignment using traditional techniques (Mok and Chung, 2020a, 2021). While these conventional methods provide high registration accuracy, they can be slow, especially for 4D fMRI images, as processing time depends on the level of misalignment. To enable faster, automated registration, some studies have explored using convolutional neural networks (CNNs) to learn both affine and deformable registration together (Huang et al., 2021; Iglesias, 2023). Many of these studies concentrate mainly on enhancing deformable registration, often treating affine registration as a basic preliminary step or neglecting it altogether (Golestani et al., 2025). As a result, the independent performance of the affine subnetwork, in comparison to existing affine registration techniques, remains unexplored. Since affine transformation deals with global alignment and large displacements, CNNs may not be the best choice for capturing image orientation and absolute position in Cartesian space (Mok and Chung, 2022).

In human brain imaging, registration benefits from specialized high-level functions such as the FSL package (Jenkinson et al., 2012) and the ANTS package (Avants et al., 2011), which are optimized for the size and spatial characteristics of the human brain. In contrast, small animal brain imaging often relies on these same high-level functions, adapting the data to fit the function parameters rather than tailoring the functions to the data. This approach can compromise data accuracy, restrict optimization possibilities, and pose significant challenges to advancing methodologies in small animal brain imaging.

While substantial progress has been made in developing robust, general-purpose preprocessing tools for human imaging data (Esteban et al., 2019), the preclinical field still lacks similarly reliable and standardized solutions. An automatic PET/MRI registration for preclinical studies based on B-splines and non-linear intensity transformation has been proposed by Bricq et al. (2018). A preclinical registration framework was introduced by Anderson et al. (2019) to address structural imaging, particularly voxel-based morphometry (VBM). A registration workflow for small animal brain MRI is proposed by Ioanas et al. (2021). However, comprehensive preprocessing pipelines that effectively handle functional preclinical data remain limited. While prior studies have proposed preclinical imaging workflows (Anderson et al., 2019; Ioanas et al., 2021), they primarily focus on structural MRI registration and analysis. In contrast, to the best of our knowledge, our proposed pipeline is the first preprocessing pipeline which integrates deep learning-based modules specifically designed for functional preclinical MRI data.

**Figure 1:**
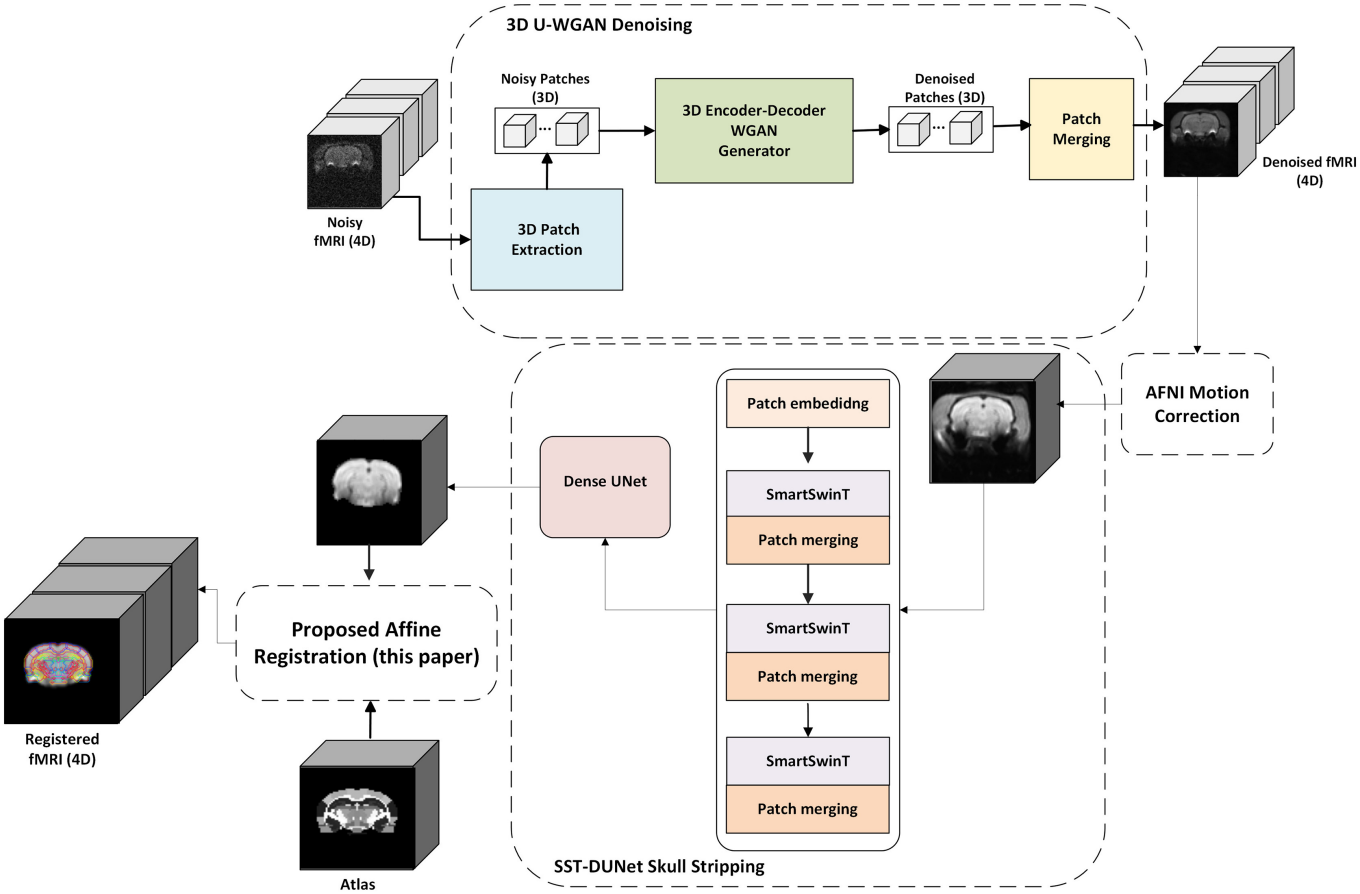
Overview of the proposed deep learning-based preclinical fMRI preprocessing pipeline. The framework integrates four key modules: (1) GAN-based denoising to suppress noise while preserving functional details, (2) motion correction using AFNI, (3) transformer-based skull stripping for accurate brain extraction, and (4) affine registration using the proposed Multi-stage Dilated Convolutional Swin Transformer (MsDCSwinT) for precise spatial alignment.

In this paper, we introduce a novel preclinical fMRI preprocessing pipeline that integrates advanced deep learning techniques, including Swin Transformer-based registration. Our pipeline incorporates our recently developed GAN-based denoising method (Soltanpour et al., 2025a), which effectively reduces noise while preserving critical functional details, and our transformer-based skull-stripping approach (Soltanpour et al., 2025b), which ensures precise brain extraction. Additionally, we propose a new affine registration method leveraging Swin Transformer (Liu et al., 2021) and dilated convolutional block (Gao et al., 2022), enabling more accurate and efficient alignment of preclinical functional MRI data. By combining these techniques, our pipeline addresses key challenges in preclinical imaging, improving data quality and facilitating more reliable downstream analyses. The general framework of the proposed pipeline is illustrated in Figure 1. The preprocessing pipeline contains four modules including our GAN-based denoising, motion correction using AFNI (Cox, 1996), our transformer-based skull stripping, and the proposed Multi-stage Dilated Convolutinal Swin Transformer (MsDCSwinT) affine registration.

The main contributions of this work are threefold. First, we present a novel end-to-end preprocessing pipeline tailored for preclinical fMRI data, which combines advanced deep learning methods to address the unique challenges of small animal brain imaging. Second, we introduce a GAN-based denoising method that effectively suppresses noise while preserving functional signal integrity, and a transformer-based skull stripping approach that ensures accurate and consistent brain extraction across subjects. Third, we propose a new affine registration framework, Multi-stage Dilated Convolutional Swin Transformer (MsDCSwinT), that improves the precision and speed of spatial alignment in preclinical fMRI datasets by leveraging hierarchical attention mechanisms and dilated convolutions. To the best of our knowledge, this is the first work to introduce a deep learning-based preprocessing pipeline specifically designed for preclinical fMRI studies. Together, these contributions offer a comprehensive and automated solution that enhances the robustness, accuracy, and efficiency of preclinical functional neuroimaging workflows.

The remainder of the paper is organized as follows: Section 2 presents the materials used in this study and introduces a novel affine registration algorithm based on a multi-stage Swin Transformer architecture. Section 3 reports the experimental results. Section 4 discusses the findings and outlines the study's limitations. Finally, Section 5 concludes the paper and outlines directions for future work.

## 2 Materials and methods

### 2.1 Datasets

This study utilized two in-house datasets comprising seven studies and a total of 280 rats, collected by the Center for Translational NeuroImaging (CTNI) at Northeastern University, Boston, MA, USA. No new animals were scanned for this work. All imaging was previously performed using a Bruker Biospec 7.0T/20-cm USR horizontal magnet with a 20-G/cm gradient insert (ID = 12 cm, 120-μs rise time), and data were acquired using built-in quad-coil electronics within the animal restrainer. Male Sprague Dawley rats (325–350 g) from Charles River Laboratories (Wilmington, MA, USA) were housed under standard 12:12 h light-dark conditions with unrestricted access to food and water. All animal procedures followed the *Guide for the Care and Use of Laboratory Animals* (NIH Publication No. 85-23, Revised 1985) and were approved by the Institutional Animal Care and Use Committee at Northeastern University, adhering to NIH and AALAS guidelines.

For each imaging session, high-resolution anatomical scans were acquired using a RARE sequence (25 slices, 1 mm thickness, FOV 3.0 cm, resolution 256 × 256, TR = 2.5 s, TE = 12.4 ms, NEX = 6, ~6 min total). Task-based fMRI data were collected using a Half-Fourier, single-shot turbo-spin echo (RARE-st) sequence with 96 × 96 in-plane resolution, 20–25 slices, TR = 6,000 ms, TE = 48 ms, RARE factor = 36, NEX = 1, repeated 100 times over a 10-min session. Resting-state fMRI (rsfMRI) data were acquired before and after the task-based scans using a spin-echo triple-shot EPI sequence (96 × 96 × [20–25 slices], voxel size = 0.312 × 0.312 mm, slice thickness = 1.2 mm, TR = 1000 ms, TE = 15 ms, 300 repetitions, ~15 min total scan time).

#### 2.1.1 Data for training and test

We apply the proposed affine model for registering rat brain functional images. We applied 6 studies (dataset 1) containing 270 samples for training and test. To train the model, we initially created a training dataset by randomly selecting 80% of the data, reserving the remaining 20% for final performance testing. During the training phase, an additional 80% of the data were randomly sampled from the training dataset, leaving the remaining 20% for validating the training of the model. This training-validation process was repeated five times to ensure an unbiased data distribution. Subsequently, the model with the highest averaged validation accuracy was chosen as the final model for testing. To evaluate the model's generalization ability, we applied one study (dataset 2), containing 10 subjects which has not been applied for training.

#### 2.1.2 Data preprocessing for affine registration

Data preprocessing before affine registration is critical for enhancing the model's robustness and generalization. We apply a standard preprocessing steps, including denoising, motion correction, and skull stripping. In our affine registration method, we align the fMRI scans to a standardized 256 × 256 × 64 atlas (Wang et al., 2025; Fuini et al., 2025). This atlas, developed by Ekam Imaging (Boston, MA, USA), provides a consistent anatomical reference that facilitates accurate spatial normalization across subjects. By registering all scans to this common space, we ensure comparability in subsequent analysis steps, enabling robust group-level statistical studies and downstream applications in functional neuroimaging. It should be noted that this step performs only affine (global) alignment rather than deformable (non-linear) registration. The proposed MsDCSwinT model estimates affine parameters (translation, rotation, scaling, and shearing) to bring each subject's fMRI volume into coarse correspondence with the standardized atlas space. This affine transformation provides a globally aligned reference frame for downstream group analyses. Deformable or non-linear alignment, which refines local anatomical correspondence, is beyond the current scope and remains part of our planned future work.

fMRI data are inherently noisy due to physiological, hardware-related, and external artifacts, making denoising a critical preprocessing step. As shown in Figure 1, we apply our 3D U-WGAN (Soltanpour et al., 2025a), a structure-preserving denoising method based on a 3D Wasserstein GAN with a 3D dense U-Net discriminator. This approach processes 4D fMRI data to retain both spatial and temporal features while effectively mitigating noise. The 3D dense U-Net discriminator captures both global and local patterns, and the inclusion of adversarial and perceptual losses helps prevent oversmoothing and preserve structural integrity. This denoising step enhances downstream processing, including affine registration, by providing cleaner and more reliable fMRI data.

Motion correction is an essential preprocessing step in fMRI analysis to reduce the impact of subject movement during image acquisition. In our pipeline, we applied AFNI's rigid-body motion correction method (Cox, 1996) prior to skull stripping to ensure temporal alignment of the brain volumes and improve the accuracy of subsequent steps.

Prior to affine registration, skull stripping as illustrated in Figure 1 is applied to remove non-brain tissues and improve anatomical alignment. Manual skull stripping is time-consuming and prone to variability, especially in preclinical fMRI data, which present challenges such as low resolution, varying slice sizes, and anatomical differences. To address these issues, we incorporate our recently developed SST-DUNet method (Soltanpour et al., 2025b), which combines a dense U-Net architecture with a Smart Swin Transformer-based feature extractor (Fu et al., 2024). The Smart Shifted Window Multi-Head Self-Attention (SSW-MSA) module enables robust feature learning by focusing on channel-wise dependencies within brain structures. Additionally, a hybrid loss function combining Focal and Dice loss mitigates class imbalance, resulting in more accurate skull extraction. This automated approach ensures reliable brain masking, which is essential for accurate affine registration.

### 2.2 Proposed affine registration algorithm

**Figure 2:**
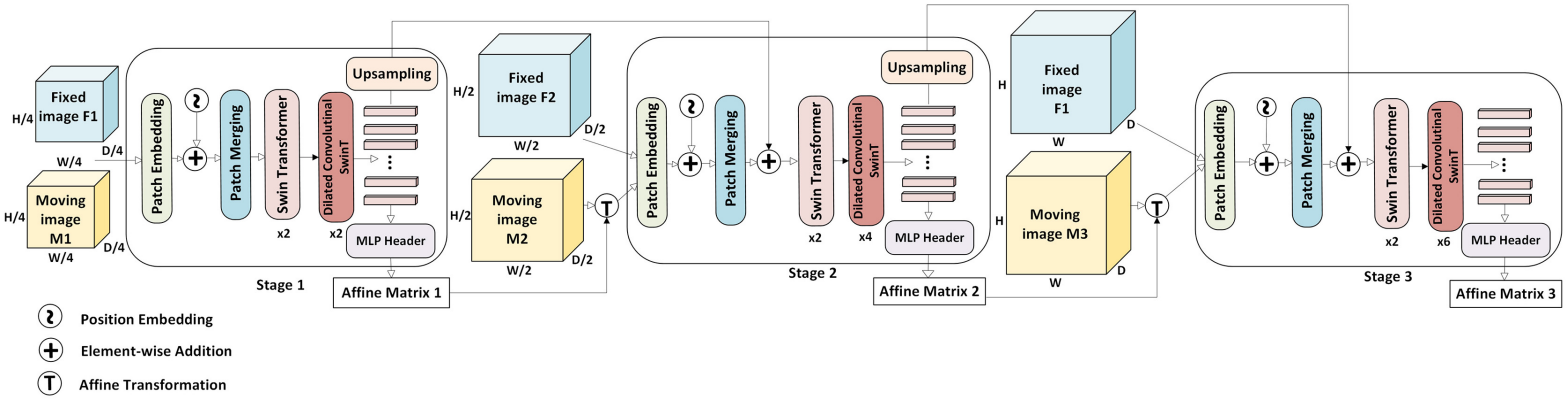
Overview of the MsDCSwinT affine registration algorithm. The model contains three stages to extract the final affine matrix.

The framework of the proposed affine registration method is presented in Figure 2. Inspired by the Coarse-to-Fine Vision Transformer (C2FViT) proposed by Mok and Chung (2022) for affine registration of clinical MRI, our method follows a multi-stage hierarchical approach to solve affine registration using an image pyramid. Unlike standard Vision Transformers (ViT) (Dosovitskiy et al., 2020), which rely on self-attention over fixed patches and struggle with local feature extraction, we incorporate Swin Transformer (SwinT) blocks (Liu et al., 2021) alongside dilated convolutional block (Gao et al., 2022) to enhance feature representation and spatial awareness.

While C2FViT utilizes convolutional patch embedding to encode local features at the input stage, it relies solely on transformer-based global attention throughout the network, lacking additional mechanisms for preserving local spatial relationships during deeper stages. In contrast, our model applies linear patch embedding followed by Swin Transformer blocks, which capture local features through windowed attention mechanisms. Importantly, we apply a dilated convolutional SwinT block after each stage, which explicitly enhances local feature modeling by expanding the receptive field without sacrificing spatial resolution. This architectural design allows our design to jointly model local anatomical variations and global structural alignments throughout all network depths, improving registration performance particularly in preclinical fMRI data. In contrast to ViT, SwinT employs a hierarchical structure to address dense prediction tasks while lowering computational costs. It achieves this by computing self-attention within non-overlapping windows of limited size. Additionally, to capture contextual details, the window configurations vary across successive layers. As a result, the network processes broader contextual information through localized self-attention mechanisms.

Our framework consists of three stages, each maintaining a similar architecture comprising a patch embedding layer, SwinT-based encoder and dilated convolutional blocks. In the first stage, a shallow encoder is employed to extract coarse structural information. In subsequent stages, the encoder depth increases to accommodate higher-resolution inputs, ensuring robust multi-scale feature learning. By leveraging the strengths of SwinT's hierarchical windowed attention alongside dilated convolutional blocks, our approach mitigates the limitations of ViT in capturing spatial dependencies and preserving the spatial integrity of functional regions, leading to more precise affine transformations for preclinical fMRI registration. Unlike the ViT, the SwinT employs a hierarchical structure that is well-suited for spatially dense tasks such as affine registration, while also reducing computational overhead. It performs self-attention within small, non-overlapping windows, allowing for efficient local feature extraction. To capture broader spatial context, the window partitions are shifted across successive layers, enabling the network to progressively model long-range dependencies through a series of local self-attention operations across the entire image volume.

In the proposed model, global spatial relationships are effectively captured through the shifted window self-attention mechanism of the Swin Transformer blocks. By progressively shifting the window partitions between layers, the model enables information flow across non-local regions, thereby modeling long-range dependencies across the full image. Additionally, the hierarchical multi-scale structure, which processes input volumes at varying resolutions, further enhances the network's ability to capture global spatial context by operating at progressively coarser scales where individual windows encompass larger anatomical regions.

Let *F* and *M* denote the fixed and moving 3D volumes, respectively, defined over a spatial domain Ω⊆ℝ^3^. This study aims to learn an optimal affine transformation matrix that aligns *F* and *M*. The affine registration is formulated as a learnable function *f*_θ_(*F, M*)=*A*, where θ represents the set of trainable parameters and *A* is the resulting affine transformation matrix. To enable multi-stage learning, an input pyramid is constructed by downsampling *F* and *M* using trilinear interpolation, yielding scaled versions *F*_*i*_∈{*F*_1_, *F*_2_, *F*_3_} and *M*_*i*_∈{*M*_1_, *M*_2_, *M*_3_}. Each *F*_*i*_ and *M*_*i*_ corresponds to a downsampled version of *F* and *M* at a scale of 0.5^(3−*i*)^.

#### 2.2.1 Patch embedding and merging blocks

For the fixed and moving images with the size of *H*×*W*×*D*, where *H, W*, and *D* are the image spatial dimension, we calculate the patch embeddings. In the first stage, where the input images have smaller resolution, we use a window of size 2 × 2 × 2. In the next stages, by increasing the resolutions, windows of size 4 × 4 × 4 and 8 × 8 × 8 are used to capture the larger patches. For the data with size *H*×*W*×*D*, we obtain vectors with the same number of features but with various feature lengths. In this way, for both *F*_*i*_ and *M*_*i*_, we extract patches with length *h*×*w*×*d*×8, *h*×*w*×*d*×64, and *h*×*w*×*d*×512 for three different stages. We employ fully connected layers to convert the variable-length feature representations into fixed-size vectors of dimension *C*, ensuring uniformity in the output dimensions. Consequently, the general feature map is defined by ${Z_{F}}^{i}\in {\mathbb{R}}^{h\times w\times d\times C}$, and ${Z_{M}}^{i}\in {\mathbb{R}}^{h\times w\times d\times C}$ for the fixes and moving images.

The patch merging operation, applied after each patch embedding and within each SwinT block, follows the same approach used in ViT. This mechanism is employed to create connections between non-overlapping image patches. Initially, the patch merging layer combines the features of adjacent 2 × 2 patches, resulting in a 4C-dimensional representation. This aggregated feature set is then passed through a linear layer, which not only reduces the number of tokens by a factor of four, equivalent to a twofold decrease in spatial resolution, but also transforms the feature representation accordingly. In this way, ${Z_{F}}^{i}$ and ${Z_{M}}^{i}$ are transformed to the same dimension as ℝ^*h*/2 × *w*/2 × *d*/2 × 2*C*^

#### 2.2.2 Dilated convolutional Swin Transformer

To further enhance the affine registration performance, we integrate the dilated convolutional block, as proposed by Gao et al. (2022), into our model. This design combines dilated convolutions with the SwinT architecture, enabling the model to capture both local and global contextual information across images. By leveraging dilated convolutions, we expand the receptive field without increasing computational complexity, which allows for more accurate localization of features in both the fixed and moving images. This is particularly advantageous for preclinical fMRI data, where image resolution and variability in structural features present significant challenges. Additionally, the SwinT's window-based self-attention mechanism facilitates capturing long-range dependencies, further improving the registration accuracy. Integrating the dilated convolutional block provides a robust approach to handling complex variations in brain geometry and resolution, ultimately enhancing the precision and reliability of the affine registration process.

In our affine Swin Transformer framework, we incorporate dilated convolution to effectively expand the receptive field across multiple stages without reducing spatial resolution. Dilated convolution proposed by Yu and Koltun (2015), enables wider spatial context modeling compared to traditional convolutions. For example, while a standard 3 × 3 × 3 kernel captures local features within a limited 3 × 3 × 3 region, a 2-dilated convolution with the same kernel expands the receptive field to 7 × 7 × 7. This expansion is particularly advantageous in our model, where 3D inputs are processed through three hierarchical stages. By using dilated convolution at each stage, we enhance the model's ability to capture global affine transformations and structural dependencies across the full 3D volume, while maintaining spatial detail and resolution.

Considering that the data flow in SwinT uses vectors instead of feature maps, as in traditional CNNs, the dilated convolution block first reshapes a group of vector features into a spatial feature map. For instance, a set of tokens with dimensions $\frac{h}{p}\times \frac{w}{p}\times \frac{d}{p}$ is reshaped into a feature map of size $\frac{h}{p}\times \frac{w}{p}\times \frac{d}{p}\times C$ where *p* is the patch size. Following this, two dilated convolutional layers with Batch Normalization (Ioffe and Szegedy, 2015) and ReLU activation are applied to capture large-range spatial features.Finally, the feature map is re-transformed back into the original token dimensions $\frac{h}{p}\times \frac{w}{p}\times \frac{d}{p}$ and passed to the next stage in the model. This integration of dilated convolutions with SwinT allows the model to capture both local and global spatial features, improving the overall accuracy and robustness of affine registration, particularly in challenging preclinical fMRI datasets.

#### 2.2.3 Affine transformation

**Figure 3:**
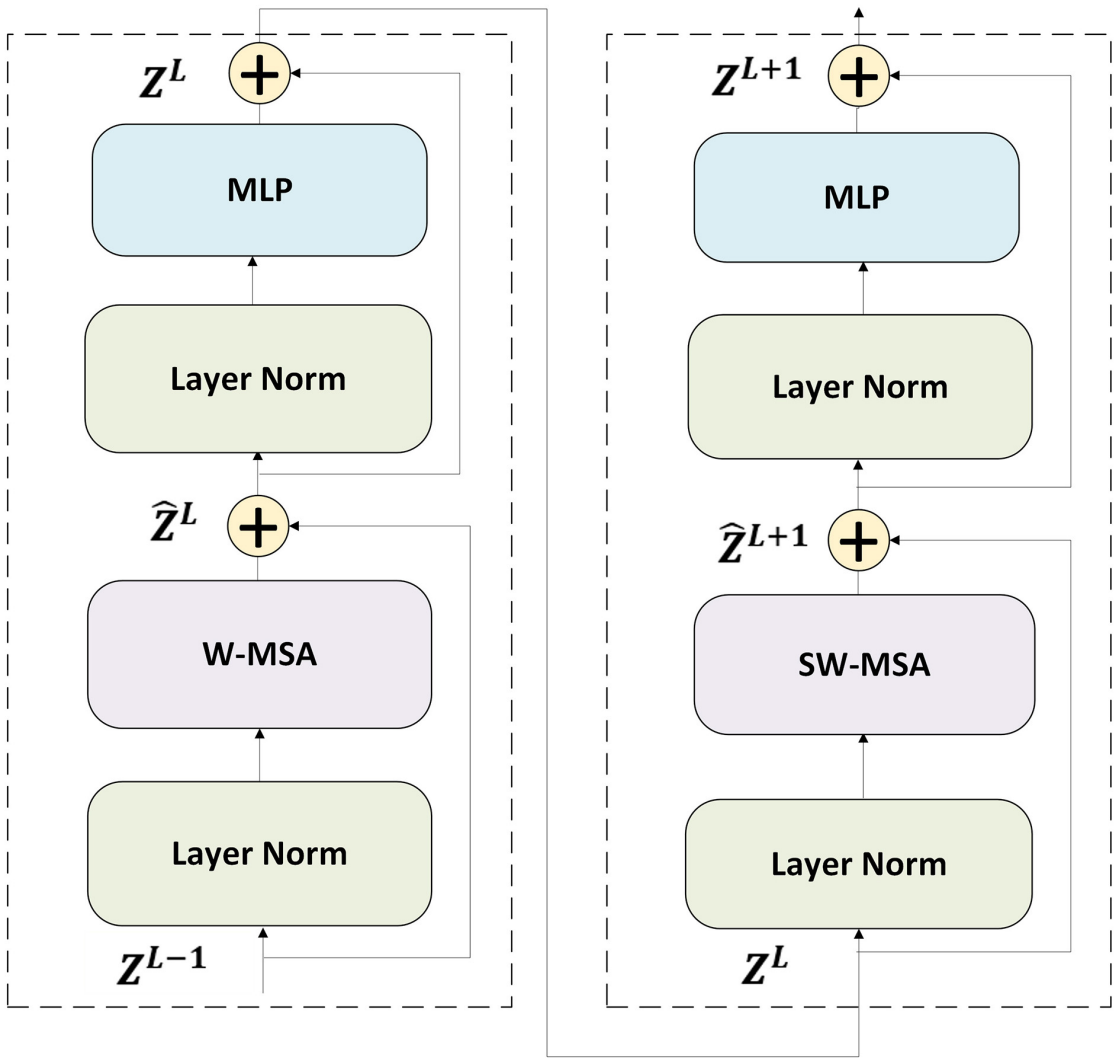
Two consecutive Swin Transformer blocks are used, incorporating W-MSA and SW-MSA, which are multi-head self-attention mechanisms employing regular and shifted window configurations, respectively.

The hierarchical self attention mechanism of the SwinT (Liu et al., 2021) is highly effective to model long range dependencies within sequences of embeddings. In our algorithm, the misalignment between fixed and moving images is captured through two consecutive SwinT blocks including W-MSA and SW-MSA which are multi-head self attention modules with regular and shifted windowing configurations, respectively. The structure of SwinT block has been shown in Figure 3.

The formula to represent the SwinT block is as follows:


$$\begin{array}{l} \begin{array}{c} {Ẑ}^{l}=W_{\text{MSA}}\left(\text{LN}\left({Ẑ}^{l-1}\right)\right)+{Ẑ}^{l-1}\\ Z^{l}=\text{MLP}\left(\text{LN}\left({Ẑ}^{l}\right)\right)+{Ẑ}^{l}\\ {Ẑ}^{l+1}=SW_{\text{MSA}}\left(\text{LN}\left(Z^{l}\right)\right)+Z^{l}\\ Z^{l+1}=\text{MLP}\left(\text{LN}\left({Ẑ}^{l+1}\right)\right)+{Ẑ}^{l+1} \end{array} \end{array}$$ (1)

where Ẑ^*l*^ and *Z*^*l*^ denote the output of block *l*. *LN* (LayerNorm) represents a regularization. *MLP* denotes multi-layer preceptron. *W*_*MSA*_ and *SW*_*MSA*_ represent the window self-attentive mechanism and the shifted window self-attentive respectively. Finally, the attention head's output is input to the dilated convultional block and the output is passed through a multi-layer linear network, which generates the corresponding affine transformation matrix *A*_*i*_.

The transformer encoders in MsDCSwinT use the similarity between projected query-key pairs to capture misalignment and global relationships between the fixed and moving images, generating attention scores for each patch embedding. The query (*Q*), key (*K*), and value (*V*) are linearly projected from the patch embeddings (tokens).


$$\begin{array}{l} Q=ẐW^{Q},K=ẐW^{K},V=ẐW^{V} \end{array}$$ (2)

Considering that the number of attention heads in the SwinT block is *h*′, the linear projection matrices are ${W_{j}}^{Q},{W_{j}}^{K},{W_{j}}^{V}\in {\mathbb{R}}^{D\times D_{h^{\prime }}}$, and $D_{h^{\prime }}=D/h^{\prime }$. Attention operation for attention head *j* is calculated as follows:


$$\begin{array}{l} \text{Attention}\left(Q_{j},K_{j},V_{j}\right)=\text{Softmax}\left(\frac{Q_{j}{K_{j}}^{T}}{\sqrt{D_{h^{\prime }}}}+B_{p}\right)V_{j} \end{array}$$ (3)

where $D_{h^{\prime }}$ is the embedding dimension and *B*_*P*_ denotes the relative position encoding. In this study, we employ *h*′=2 attention heads for all the transformer encoders. Finally, the attended embeddings from all attention heads are concatenated and passed through a linear projection matrix.

#### 2.2.4 Multi-stage affine transformation estimation

We incorporate a multiresolution approach into our architecture. Specifically, each stage of MsDCSwinT includes a classification head, which consists of two consecutive MLP layers with a hyperbolic tangent (Tanh) activation function. This classification head processes the averaged patch-wise embeddings and generates a set of affine parameters. At each intermediate stage *i*, the resulting affine matrix is applied to progressively transform the moving image *M*_*i*+1_ via a warping operation using a spatial transformer (Jaderberg et al., 2015). The warped image *M*_*i*+1_ is then concatenated with the fixed image *F*_*i*+1_ and passed to the next stage, *i*+1. This progressive transformation strategy allows for initial misalignments to be corrected at lower resolutions, enabling higher-level transformers to focus on more complex misalignments, thus simplifying the problem at later stages.

#### 2.2.5 Geometric transformation model

Rather than directly estimating the affine transformation matrix, our model predicts a set of geometric transformation parameters. Specifically, the affine registration problem is reformulated as:


$$\begin{array}{l} f_{\theta }\left(F,M\right)=\left[\text{t},\text{r},\text{s},\text{h}\right] \end{array}$$ (4)

We parameterize the affine transform with [*t, r, s, h*]∈ℝ^12^, where *t*=(*t*_*x*_, *t*_*y*_, *t*_*z*_), *r*=(*r*_*x*_, *r*_*y*_, *r*_*z*_), *s*=(*s*_*x*_, *s*_*y*_, *s*_*z*_), and *h*=(*h*_*xy*_, *h*_*xz*_, *h*_*yz*_). Using homogeneous coordinates, the overall affine matrix **A** is the ordered product:


$$\begin{array}{l} \text{A}=\text{T}\cdot \text{R}\cdot \text{S}\cdot \text{H} \end{array}$$ (5)

where *T*, *R*, *S*, and *H* represent translation, rotation, scaling, and shearing matrices, respectively.

Translation (*T*):


$$T=\left[\begin{array}{cccc} 1 & 0 & 0 & t_{x}\\ 0 & 1 & 0 & t_{y}\\ 0 & 0 & 1 & t_{z}\\ 0 & 0 & 0 & 1 \end{array}\right]$$

Rotation (*R*): The overall rotation matrix is defined as:


$$R=R_{x}\left(r_{x}\right)R_{y}\left(r_{y}\right)R_{z}\left(r_{z}\right)$$

Rotation about *x*-axis:


$$R_{x}=\left[\begin{array}{cccc} 1 & 0 & 0 & 0\\ 0 & \cos r_{x} & -\sin r_{x} & 0\\ 0 & \sin r_{x} & \cos r_{x} & 0\\ 0 & 0 & 0 & 1 \end{array}\right]$$

Rotation about *y*-axis:


$$R_{y}=\left[\begin{array}{cccc} \cos r_{y} & 0 & \sin r_{y} & 0\\ 0 & 1 & 0 & 0\\ -\sin r_{y} & 0 & \cos r_{y} & 0\\ 0 & 0 & 0 & 1 \end{array}\right]$$

Rotation about *z*-axis:


$$R_{z}=\left[\begin{array}{cccc} \cos r_{z} & -\sin r_{z} & 0 & 0\\ \sin r_{z} & \cos r_{z} & 0 & 0\\ 0 & 0 & 1 & 0\\ 0 & 0 & 0 & 1 \end{array}\right]$$

Scaling (*S*):


$$S=\left[\begin{array}{cccc} s_{x} & 0 & 0 & 0\\ 0 & s_{y} & 0 & 0\\ 0 & 0 & s_{z} & 0\\ 0 & 0 & 0 & 1 \end{array}\right]$$

Shearing (*H*):


$$H=\left[\begin{array}{cccc} 1 & h_{xy} & h_{xz} & 0\\ 0 & 1 & h_{yz} & 0\\ 0 & 0 & 1 & 0\\ 0 & 0 & 0 & 1 \end{array}\right]$$

To reduce the search space and enforce meaningful transformations, we constrain the predicted parameters during training as follows: rotation and shearing values are limited to the range [−π, π], translation values are constrained within ±50% of the image resolution, and scaling values are restricted to the range [0.5, 1.5]. Additionally, we apply the center of mass of the image **c**_*I*_ (Mok and Chung, 2022) computed as:


$$\begin{array}{l} {\text{c}}_{I}=\frac{\sum_{p\in \text{Ω}}p\cdot I\left(p\right)}{\sum_{p\in \text{Ω}}I\left(p\right)} \end{array}$$ (6)

where Ω represents the spatial domain of the image and *p* denotes the voxel position.

#### 2.2.6 Loss function

Our affine registration method is based on unsupervised learning. This is primarily because generating manual segmentation maps for fMRI data is extremely time-consuming and impractical. Additionally, creating manual maps using the mean of fMRI data often results in inconsistent segmentation, particularly due to the low resolution of the data, which can lead to varying numbers of detected regions across different samples. Given these challenges and the unavailability of reliable ground truth labels, we opted for an unsupervised approach that does not rely on manually annotated data. Instead, the model learns to align the input image with an atlas by optimizing a similarity measure between them, allowing for robust and automated registration even in the absence of labelled training data.

The affine registration problem is parametrized as a learning problem to minimize the following equation:


$$\begin{array}{l} \theta^{*}=argmin_{\theta }\left[{𝔼}_{\left(F,M\right)\in D}L\left(F,M\left(\phi \left(A\right)\right)\right)\right] \end{array}$$ (7)

where θ represents the learning parameters in the MsDCSwinT model, *F* is the atlas, and *M* is the moving image from the training dataset *D*. The loss function L measures the similarity between the atlas *F* and the affine transformed moving image *M*(ϕ(*A*)). We use the negative Normalized Cross-Correlation (NCC) (Mok and Chung, 2020b) similarity measure to quantify the distance between *F* and *M*(ϕ(*A*)) as follows:


$$\begin{array}{l} L_{\text{sim}}\left(F,M\left(\phi \right)\right)=\underset{i\in \left[1..L\right]}{\sum }-\frac{1}{2^{\left(L-i\right)}}{\text{NCC}}_{w}\left(F_{i},M_{i}\left(\phi \right)\right) \end{array}$$ (8)

where *L* represents the number of image pyramid levels, *NCC*_*w*_ denotes the local normalized cross-correlation with window size *w*×*w*, and (*F*_*i*_, *M*_*i*_) are the atlas and moving images in the image pyramid.

The overall loss function is inspired by the energy-based formulation used in traditional image registration. It consists of two components: a similarity loss between the fixed image (atlas) and the affine-transformed moving image, and a regularization term that constrains the affine parameters to prevent implausible transformations. The total loss is defined as:


$$\begin{array}{l} L\left(F,M,\phi \right)=L_{\text{sim}}\left(F,M\left(\phi \right)\right)+\lambda R\left(\phi \right) \end{array}$$ (9)

where *F* is the fixed image (atlas), *M* is the moving image, and ϕ denotes the affine transformation predicted by the network. The similarity term $L_{\text{sim}}$ quantifies alignment quality, for which we use a multi-resolution negative normalized cross-correlation (NCC) as described in Equation 8.

The regularization term R(ϕ) penalizes overly large or unrealistic affine transformations to maintain stable optimization. In our work, we regularize the rotation and shearing parameters by constraining them within [−π, +π], translation within [−0.5*R*, +0.5*R*], and scaling between [0.5, 1.5], where *R* denotes the spatial resolution of the input image. This encourages the model to learn physically plausible transformations while maintaining registration accuracy. Here, λ is a regularization weighting parameter that balances the contribution of the similarity loss and the regularization term. A higher value of λ enforces stronger constraints on the transformation parameters, while a lower value focuses more on image similarity. In our experiments, we empirically set λ=0.01 to achieve a good balance between accuracy and stability.

#### 2.2.7 Implementation details

We applied the mean of fMRI scans to create 3D MRI data. We resampled and padded all scans to 96 × 96 × 48 with the same resolution (0.3mm × 0.3mm × 0.4mm). The input voxel values were adjusted to fall within the range of 0.0–1.0 through normalization. The model was implemented using the PyTorch framework and trained on an Nvidia GeForce RTX 4090 GPU with CUDA support. We used the Adam optimizer with a fixed learning rate of 0.0001 and a batch size of 1. The value of λ=0.01 was selected based on validation performance to ensure optimal registration accuracy without overfitting the transformation parameters.

### 2.3 Functional connectivity analysis

#### 2.3.1 Preprocessing

All fMRI datasets were preprocessed using AFNI (https://afni.nimh.nih.gov/). Each image volume was smoothed using a Gaussian kernel with a full width at half maximum (FWHM) of 0.1 mm. The data were then cropped using a previously generated brain mask, aligned to a down-sampled rat 96 × 96 × 48 template space, and band-pass filtered within the frequency range of 0.01–0.1 Hz to remove physiological and low-frequency noise artifacts. These steps follow established preprocessing protocols (Lupinsky et al., 2025; Sourty et al., 2024b,a; Nasseef et al., 2021; Karatas et al., 2021).

#### 2.3.2 Post-processing and functional connectivity analysis

Post-processing included seed-based functional connectivity (FC) analysis, one of the most widely adopted methods in rodent fMRI studies (Lupinsky et al., 2025; Sourty et al., 2024a,b; Nasseef et al., 2021; Karatas et al., 2021; Nasseef et al., 2019). Seed-to-seed correlation analysis was conducted on datasets from eight rats, all of which underwent identical preprocessing pipelines, except for the registration methods. Based on these methods, five registration groups were established including MsDCSwinT (ours), ANTS, C2FViT, C2FGALF, and ConvNet.

#### 2.3.3 Atlas-based region definition

To define anatomical brain regions, we utilized a previously generated, in-house high-resolution rat brain atlas (Wang et al., 2025; Fuini et al., 2025), initially composed of 176 brain regions at a spatial resolution of 256 × 256 × 64. To ensure compatibility between the lower-resolution functional imaging data and the high-resolution rat brain atlas, the atlas was down-sampled to a spatial resolution of 96 × 96 × 48, yielding a total of 159 functional brain regions.

#### 2.3.4 Seed mask construction and connectivity computation

Seed regions were defined based on the down-sampled atlas. The mean time series for each seed region was extracted, and pairwise Pearson correlations were computed and Fisher's Z transformation was applied to generate the seed-to-seed FC matrix. Statistical significance of connectivity differences between groups was assessed using two-sample t-tests with False Discovery Rate (FDR) correction (Nasseef et al., 2021, 2019, 2018). Statistical significance of connectivity differences between groups was assessed using two-sample t-tests with False Discovery Rate (FDR) correction (Nasseef et al., 2021, 2018, 2019)). MATLAB-based Multiple Testing Toolbox (https://www.mathworks.com/matlabcentral/fileexchange/70604-multiple-testing-toolbox) was used for circular plotting and FDR correction. All computations were carried out using an in-house developed MATLAB script, following our previously validated pipeline (Lupinsky et al., 2025; Nasseef et al., 2021, 2019).

## 3 Experimental results

### 3.1 Affine registration performance analysis

To evaluate the performance of our proposed MsDCSwinT model in preclinical fMRI affine registration, a comparative analysis was conducted against existing methods. The evaluated methods included an iterative technique implemented in ANTS (Avants et al., 2011), and learning-based affine methods ConvNet (De Vos et al., 2019), C2FViT (Mok and Chung, 2022), and C2FGALF (Ji and Yang, 2024) developed for clinical image registration.

We use the affine registration implementation provided in the publicly available ANTS software package, which adopts a three-level multi-resolution optimization framework based on adaptive gradient descent and mutual information as the similarity metric. For learning-based methods, we follow the parameter settings recommended in their respective publications. All models are trained in an unsupervised manner using the similarity defined in Equation 8.

To enable robust affine registration of 4D fMRI data, we first reduced the dimensionality of the training inputs by computing the mean across the time dimension. Specifically, for each 4D fMRI dataset, we averaged the voxel intensities over all time points to generate a representative 3D MRI volume. This 3D mean image captures the essential anatomical structure while reducing the impact of temporal fluctuations, facilitating more stable training of the registration network. During inference, the trained network was applied to the 3D mean image of each subject in the CTNI fMRI test set to estimate the corresponding affine transformation matrix. This affine matrix was applied uniformly to register all individual 3D volumes across the 295 time points of the 4D fMRI data. This approach ensures consistent spatial alignment throughout the entire fMRI time series.

#### 3.1.1 Evaluation metrics

To evaluate the performance of our affine registration algorithm, we align each subject's image to the atlas. The registration accuracy is assessed using the Dice Similarity Coefficient (DSC) (Dice, 1945), which quantifies the overlap between the transformed moving image and the atlas. This provides a measure of segmentation accuracy. In addition to DSC, we compute the 95% percentile of the Hausdorff Distance (HD95) (Huttenlocher et al., 1993), which measures the distance between the boundaries of the transformed moving image and the atlas. Together, these metrics offer a comprehensive view of the registration's precision and reliability.

The Dice Similarity Coefficient (DSC) for the subcortical segmentation map can be formulated as:


$$\begin{array}{l} \text{Dice}\left(S_{M\left(\phi \right)}^{k},S_{F}^{k}\right)=\frac{2|S_{M\left(\phi \right)}^{k}\cap S_{F}^{k}|}{\left|S_{M\left(\phi \right)}^{k}|+|S_{F}^{k}\right|} \end{array}$$ (10)

where $S_{M\left(\phi \right)}^{k}$ represents the set of voxels of structure *k* in the registered moving image, and $S_{F}^{k}$ represents the set of voxels of structure *k* in the fixed image (atlas).

Additionally, we evaluate the 95th percentile of the Hausdorff distance (HD95) between segmentation maps to assess the robustness of the registration algorithm. The calculation can be formulated as:


$$\begin{array}{l} {\text{HD}}_{95}\left(S_{M\left(\phi \right)}^{k},S_{F}^{k}\right)=95\%\left(d\left(S_{M\left(\phi \right)}^{k},S_{F}^{k}\right)\;||\;d\left(S_{F}^{k},S_{M\left(\phi \right)}^{k}\right)\right) \end{array}$$ (11)

where


$$d\left(S_{M\left(\phi \right)}^{k},S_{F}^{k}\right)=\left\{\underset{b\in S_{F}^{k}}{\min}||a-b{||}_{2}|a\in S_{M\left(\phi \right)}^{k}\right\},$$

$$d\left(S_{F}^{k},S_{M\left(\phi \right)}^{k}\right)=\left\{\underset{a\in S_{M\left(\phi \right)}^{k}}{\min}||b-a{||}_{2}|b\in S_{F}^{k}\right\},$$

and ||·||_2_ denotes the Euclidean distance.

In addition to reporting the traditional overall Dice Similarity Coefficient (DSC) to evaluate our model's performance, we further assessed the registration accuracy by analyzing the distribution of results. Specifically, we computed the 30% lowest DSC of all cases (DSC30), based on the registration accuracy ranking across the 159 segmented structures in the test set. To provide a more complete analysis, we also reported the DSC for unregistered data (moving data). Together, these evaluations offer a comprehensive understanding of the registration accuracy and robustness of the proposed method.

#### 3.1.2 Quantitative and qualitative affine registration evaluation results

The overall experimental results are summarized in Table 1. Our proposed method, MsDCSwinT, achieves a DSC of 0.9286 and 0.9214 on the CTNI dataset1 and dataset 2, demonstrating the best registration performance among all methods compared. The most competing conventional method, ANTS, achieves a DSC of 0.9159 and 0.9082 on dataset 1 and dataset 2 respectively. Other learning-based methods, including ConvNet, C2FViT, and C2FGALF achieve DSCs of 0.7469, 0.8408, and 0.8638 for dataset 1, and 0.7294, 0.8231, and 0.8469 for dataset 2 respectively. In comparison, our model, MsDCSwinT, not only achieves an overall registration accuracy of 0.93 but also obtains the highest DSC30 and lowest HD95 outperforming all competing methods on both datasets.

**Table 1:** Quantitative evaluation of the results on the CTNI dataset.

**Method**	**DSC**	**DSC30**	**HD95**
**Dataset1**			
Unregistered	0.4883 ± 0.1274^*^	0.3317 ± 0.0437^*^	18.9103 ± 1.2315^*^
ANTS	0.9159 ± 0.0329	0.8949 ± 0.0233^*^	2.0604 ± 0.3152
ConvNet	0.7469 ± 0.0687^*^	0.6066 ± 0.0501^*^	8.4757 ± 0.5335^*^
C2FViT	0.8408 ± 0.0514^*^	0.7315 ± 0.0459	5.1041 ± 0.5818^*^
C2FGALF	0.8638 ± 0.0470^*^	0.7627 ± 0.0402	4.8079 ± 0.4973^*^
MsDCSwinT (ours)	**0.9286** **±0.0347**	**0.9198** **±0.0287**	**1.9535** **±0.3973**
**Dataset2**			
Unregistered	0.4625 ± 0.1291^*^	0.3252 ± 0.0554^*^	19.3821 ± 1.3872^*^
ANTS	0.9082 ± 0.0334	0.8983 ± 0.0261^*^	2.7472 ± 0.3227
ConvNet	0.7294 ± 0.0702^*^	0.5901 ± 0.0613^*^	8.7963 ± 0.5741^*^
C2FViT	0.8231 ± 0.0527^*^	0.7184 ± 0.0547^*^	5.3264 ± 0.6228^*^
C2FGALF	0.8496 ± 0.0565	0.7519 ± 0.0498	5.0437 ± 0.5081^*^
MsDCSwinT (ours)	**0.9214** **±0.0551**	**0.9027** **±0.0292**	**2.1129** **±0.2036**

Metric values are reported for the registered mean of fMRI data as mean ± standard deviation.

The bolded numbers indicate the highest scores for each metric. Statistical analysis compares the performance of the proposed MsDCSwinT with other competing methods using a two-sided *t*-test, where ^*^p-value < 0.05 indicates statistical significance.

**Figure 4:**
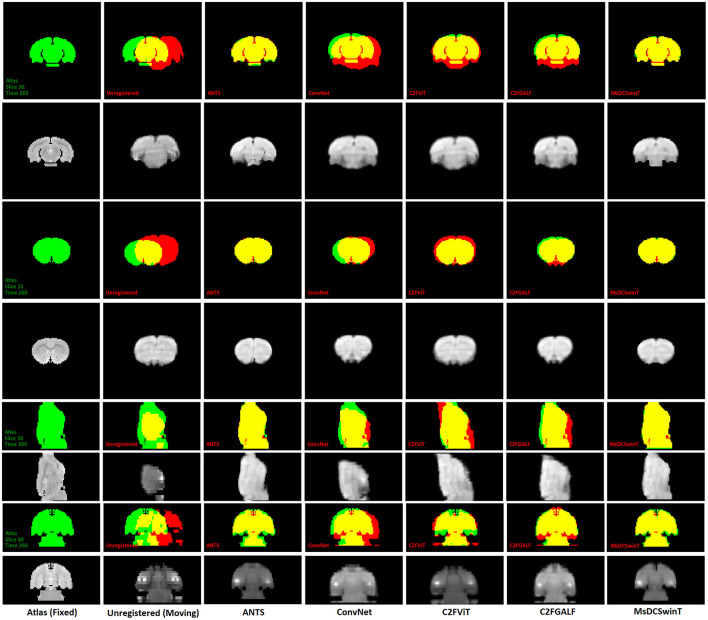
Example coronal, axial, and sagittal fMR slices from the test dataset 1 are shown. The slices are taken from the fixed image (Atlas), moving images (Unregistered), and the resulting warped images produced by ANTS, ConvNet, C2FViT, C2FGALF, and our proposed MsDCSwinT. Atlas-only regions are shown in green, registered-only regions in red, and overlapping regions in yellow, indicating alignment between the atlas and registered image.

**Figure 5:**
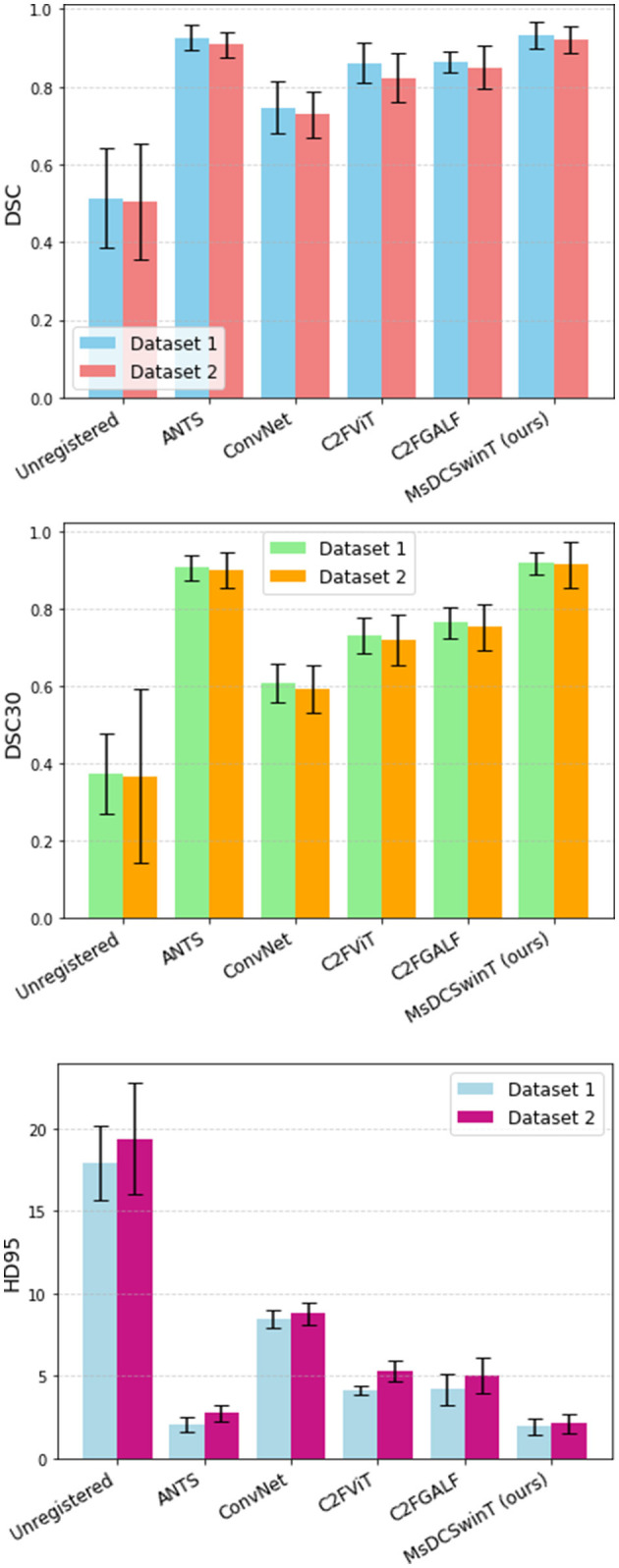
Methods comparison on CTNI fMRI test dataset 1 and dataset 2. MsDCSwinT approach is superior than the alternative approaches in terms of DSC, DSC30, and HD95.

Figure 4 presents a qualitative comparison for the test set of fMRI dataset 1, highlighting the visual differences between the moving (unregistered) image, the registered images using different methods, and the atlas across representative slices and time points. As shown in Figure 4, example coronal, axial, and sagittal slices from the test dataset 1 illustrate the visual alignment performance of various registration methods, including ANTS, ConvNet, C2FViT, C2FGALF, and our proposed MsDCSwinT model. The figure displays the fixed image (Atlas), unregistered images, and the corresponding warped outputs produced by each method. Color-coded overlays help visualize registration quality, green indicates atlas-only regions, red shows registered-only regions, and yellow highlights overlapping areas, which represent perfect alignment. Visually, our proposed MsDCSwinT achieves the highest degree of overlap with the atlas, indicated by more extensive yellow regions. While the performance of MsDCSwinT is very close to ANTS, a widely regarded traditional method, it clearly outperforms other deep learning-based approaches. These results demonstrate that our model effectively combines the robustness of traditional methods with the efficiency and automation of deep learning, achieving accurate registration while maintaining anatomical integrity. The distribution of evaluation metrics for CTNI fMRI test sets is shown in Figure 5. All results have been computed as the mean ± standard deviation over all slices and time points of the fMRI test sets.

Table 2 presents the average inference times for all methods. We report the average registration time to register the mean of fMRI data to the atlas. As the table shows our method is the fastest among the methods evaluated, mainly due to GPU acceleration and efficient learning-based design. ConvNet, C2FViT, and C2FGALF are also significantly faster than ANTS. ANTS shows variable runtimes depending on the level of initial misalignment.Our model reduces the inference time to just 0.12 s, making it much more suitable for the practical demands of preclinical image registration.

**Table 2:** Performance comparison.

**Methods**	**Parameters (M)**	**Inference time (s)**
ANTS	-	33.38 ± 2.24
ConvNet	14.8	0.21 ± 0.05
C2FViT	16.4	0.27 ± 0.08
C2FGALF	15.7	0.16 ± 0.1
MsDCSwinT (Ours)	15.2	**0.12** **±0.03**

The inference time is measured as the average runtime across results in the test set of dataset 1. The bold value indicates the best result.

To provide a complete view of the computational efficiency, we report the total preprocessing time for each subject, which includes denoising, skull stripping, and affine registration. The full proposed pipeline requires approximately 24.85 s per subject, consisting of 24.18 s for denoising, 0.55 s for skull stripping, and about 0.12 s for affine registration. For comparison, ANTs registration alone takes about 33.38 s, which is already longer than the entire runtime of our full preprocessing workflow. These results show that our proposed registration method contributes less than 1% of the total processing time and that the overall pipeline remains substantially faster than ANTs-based preprocessing approaches.

### 3.2 Functional connectivity analysis results

#### 3.2.1 Data visualization

To facilitate group-level interpretation, the results were visualized using MATLAB's built-in plotting tools. Visualization formats included scatter plots, and box plots. For circular plotting, the CircularGraph (https://www.mathworks.com/matlabcentral/fileexchange/48576-circulargraph) toolkit was applied. These representations provided intuitive insights into group-wise variability, inter-regional correlation patterns, and overall data distribution, thereby enhancing the interpretability of the findings.

#### 3.2.2 Seed-to-Seed rs-fMRI reveals high similarity between proposed MsDCSwinT and other registration methods

**Figure 6:**
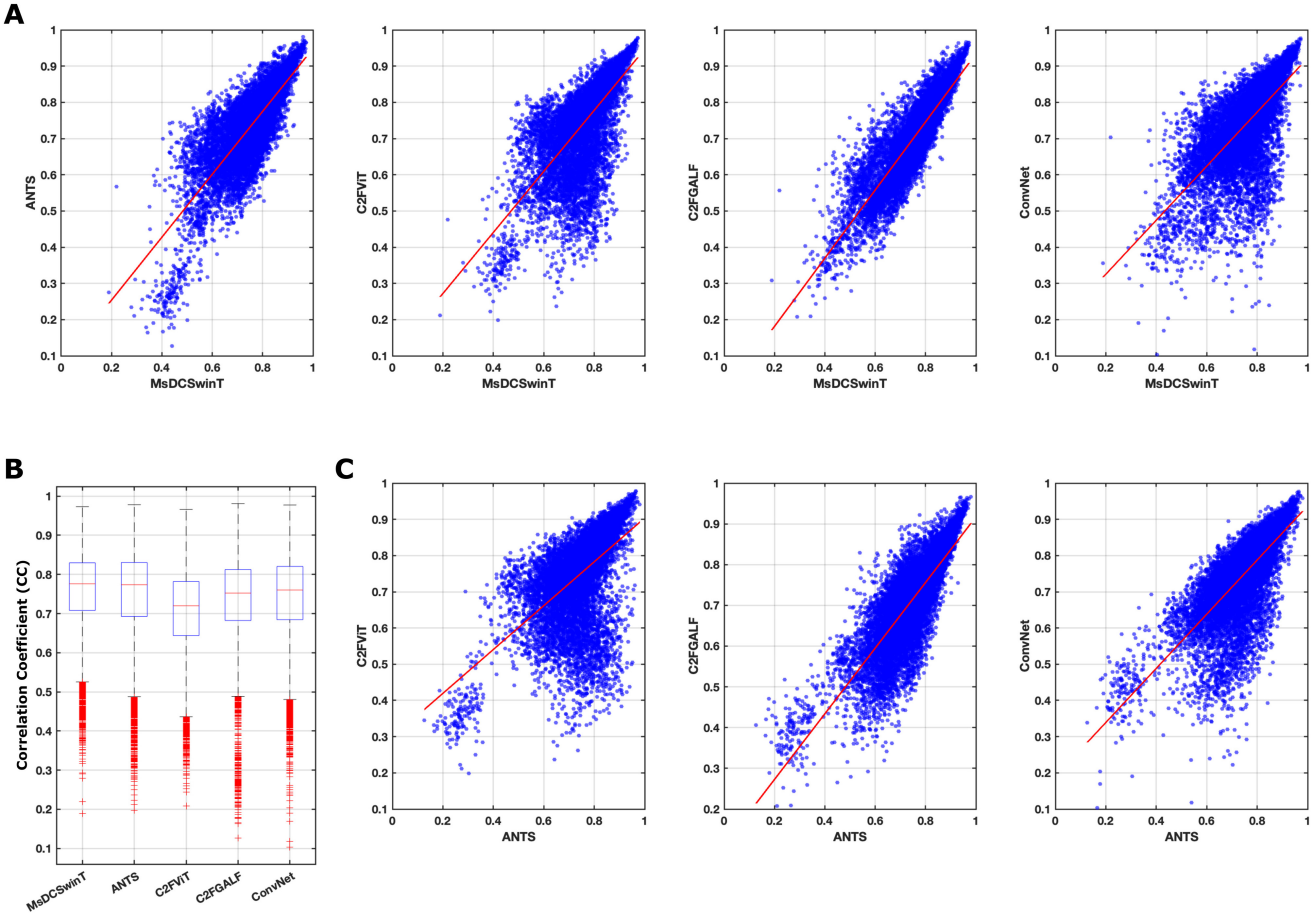
Inter-group comparison of 159-seed functional connectivity across five automated registration methods in rats; **(A)** Scatter Plot Comparison with MsDCSwinT: The four panels illustrate pairwise comparisons of functional connectivity values between the proposed MsDCSwinT method and each of the other automated four methods: ANTS, C2FViT, C2FGALF, and ConvNet. Each scatter plot displays 1,256 correlation coefficient (CC) values with fitted linear regression lines (in red), representing the distribution and degree of correspondence between methods; **(B)** Box Plot of Mean Correlation Coefficients: This panel presents the distribution of mean CC values (*n* = 1,256) for each automated registration method including our MsDCSwinT. The box plots highlight the central tendency, interquartile range, and variability across methods, facilitating inter-method comparison of overall registration performance; **(C)** Scatter Plot Comparison with ANTs: Similar to **(A)**, these three panels display pairwise comparisons between classical ANTS identified as the best-performing baseline so far and the remaining three automated methods (C2FViT, C2FGALF, and ConvNet) based on 1,256 CC values each. Regression lines (in red) indicate linear trends in performance similarity.

Resting-state fMRI (rs-fMRI) data from all eight rats, preprocessed through five different automated registration methods (including the proposed MsDCSwinT), were analyzed across 159 functional brain regions. For each dataset, mean time series were extracted from each region based on the corresponding registration. This hypothesis-driven, atlas-based approach allowed for a comprehensive whole-brain investigation of connectivity differences between the MsDCSwinT method and the five other registration strategies. To evaluate inter-group connectivity patterns, symmetric Pearson's correlation coefficients (CC) were first computed for all pairs of the 159 regions within each rat and automated registration group. The results were visualized through scatter plots comparing each method with MsDCSwinT (Figure 6A), box plots of mean CC distributions across methods (Figure 6B), and scatter plots comparing each method with the classical ANTS method (Figure 6C). The box plot analysis revealed a striking similarity in mean, quartiles, and outlier patterns between the proposed MsDCSwinT method and the classical ANTS method (Figure 6B). Similarly, scatter plots with fitted regression lines (Figure 6A, first panel) demonstrated a high degree of correlation between MsDCSwinT and ANTS, reinforcing the consistency of these findings. In contrast, comparatively lower similarity was observed between MsDCSwinT and the other three existing deep learning powered methods: C2FViT, C2FGALF, and ConvNet as shown in both scatter plots (Figure 6A, second to fourth panels) and box plots (Figure 6B). Interestingly, a similar pattern of reduced similarity was also observed when comparing ANTS with C2FViT, C2FGALF, and ConvNet (Figures 6B, C), suggesting consistent differentiation across methods.

**Figure 7:**
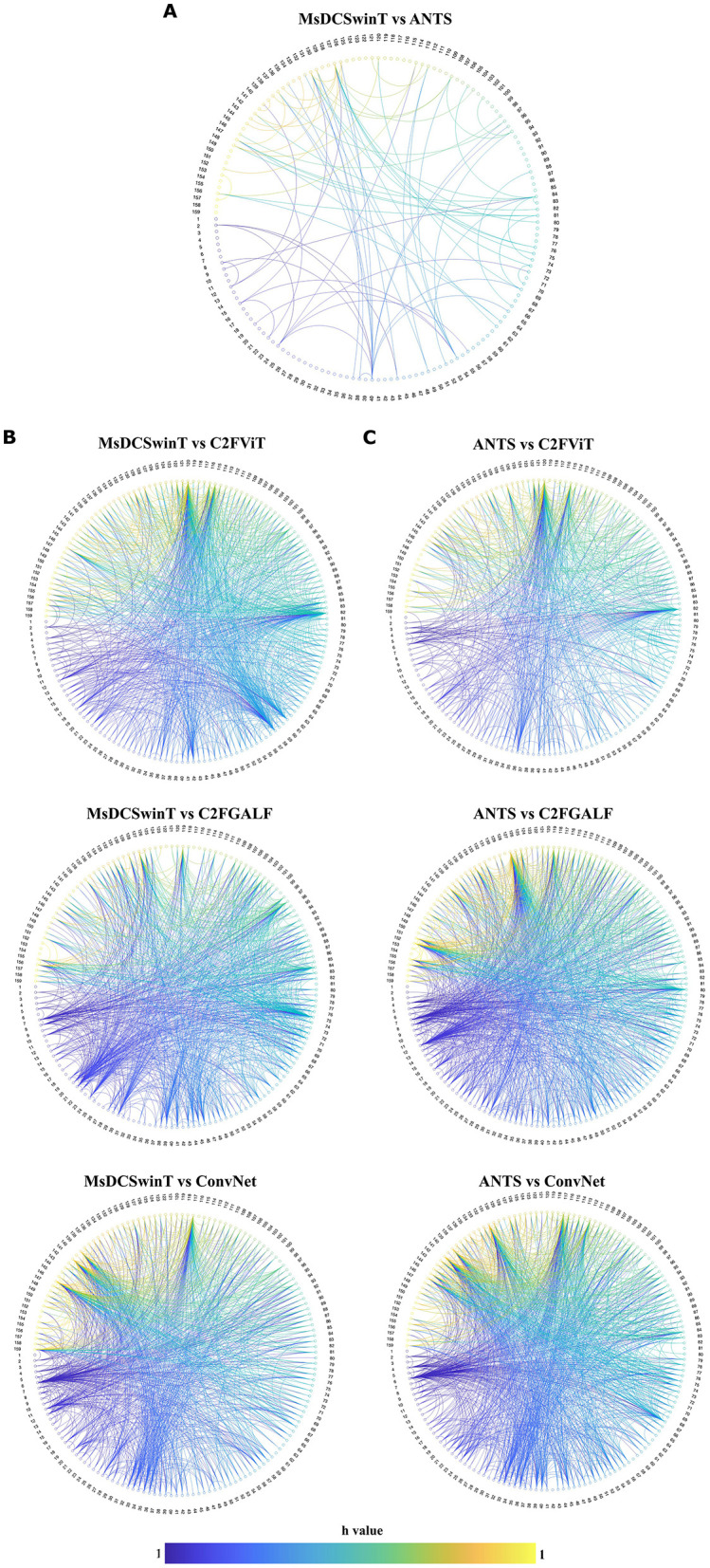
Inter-group statistical significance comparison of 159-seed functional connectivity across five automated registration methods in rats. Each panel illustrates the significant h values obtained by pairwise two-tailed *t*-test (*p* = 0.05, FDR-corrected, *n* = 8 per group), **(A, B)** comparing the proposed MsDCSwinT method to each of the four other automated registration methods, **(C)** comparing the gold standard ANTS to each of the three other automated AI registration methods across all 1,256 seed-to-seed correlation coefficient (CC) values. Statistically significant differences between node pair are represented using parula color bar with each value 1; **(A)** MsDCSwinT vs. ANTS; **(B)** top: MsDCSwinT vs. C2FViT, middle: MsDCSwinT vs. C2FGALF, bottom: MsDCSwinT vs. C2FGALF; **(C)** top: ANTS vs. C2FViT, middle: ANTS vs. C2FGALF, bottom: ANTS vs. C2FGALF; This analysis highlights method-specific statistically significant variations in alignment performance, as reflected in functional connectivity outcomes derived from atlas-based seed-to-seed correlation analysis.

To further assess group-level differences, a pairwise t-test (*p* = 0.05, two-tailed, FDR-corrected, *n* = 8 per group) was conducted to compare the proposed MsDCSwinT method with each of the four other automated registration methods respectively across all 1,256 seed-to-seed correlation coefficient (CC) values (Figure 7). Notably, very few statistically significant differences were observed between the MsDCSwinT method and the classical ANTS method (Figure 7A), indicating a high degree of similarity in their resulting connectivity patterns. In contrast, a substantially greater number of connectivity differences were identified when MsDCSwinT was compared to the other three AI-powered methods: C2FViT, C2FGALF, and ConvNet (Figure 7B), suggesting more variability in their alignment outcomes. To contextualize these results, additional comparisons between gold standard ANTS and each of the three AI methods were performed independently (Figure 7C). Visual inspection suggests that the patterns of differences for C2FGALF and ConvNet were comparable to those observed in the MsDCSwinT vs. ANTs analysis (Figures 7B, C, middle and bottom panels), while ANTS appeared to outperform MsDCSwinT slightly in the comparison with C2FViT(Figures 7B, C, top panels). Collectively, these findings reinforce that MsDCSwinT achieves connectivity profiles closely aligned with those of the widely recognized ANTS method, supporting its reliability, robustness, and suitability as a fully automated solution for preprocessing in rodent resting-state fMRI studies. Consequently, the results underscore the potential of MsDCSwinT for fully automated and more accurate rs-fMRI preprocessing and analysis.

## 4 Discussion

### 4.1 Affine registration analysis

The results show that our method performs similarly to the traditional method (ANTS), and outperforms the learning-based methods like ConvNet, C2FViT, and C2FGALF. Importantly, our method improves atlas-based registration on both CTNI fMRI datasets. Since dataset 2 was not used during training, the strong performance on it demonstrates that our method generalizes well to unseen fMRI data. Our proposed MsDCSwinT method outperforms both C2FViT and C2FGALF in terms of registration accuracy and inference speed. C2FViT introduced a coarse-to-fine vision transformer architecture to model long-range dependencies for affine registration but showed limited capability in effectively capturing multi-scale features. C2FGALF improved upon this by using multiscale convolutional kernels and a weighted global positional attention mechanism to better fuse global and local feature mappings. However, C2FGALF still faces challenges in optimally balancing fine-scale and global-scale information. In contrast, our method integrates dilated convolutions, allowing MsDCSwinT to capture local anatomical features while the transformer layers handle the global alignment. The combination of dilated convolutions and Swin Transformers in a multi-stage framework ensures that both local and global misalignments are addressed progressively, which is essential for accurate registration in fMRI data. The multi-stage approach and the Swin Transformer's ability to handle multi-scale feature extraction further improve its robustness compared to C2FViT and C2FGALF, making MsDCSwinT a more effective solution for the complex challenges of preclinical fMRI registration.

The proposed method not only matches the registration accuracy of traditional algorithm, ANTS, but also offers a substantial improvement in average inference time. Specifically, our affine model achieves significantly faster inference compared to traditional method (ANTS) and performs comparably to learning-based affine registration approaches such as ConvNet, C2FViT, and C2FGALF.

Our method uses the global connectivity of the self-attention operator while controlling the locality of the convolutional feed-forward layer. This allows it to capture global orientations, spatial positions, and long-term dependencies between the image pair to compute a set of geometric transformation parameters. Extensive experiments show that our method outperforms existing techniques, and is robust when tested on unseen dataset. It also performs slightly better than conventional method ANTS in term of registration accuracy and maintains the runtime advantages of learning-based approaches.

#### 4.1.1 Limitations

Despite its promising results, our proposed method has several limitations. It is currently tailored for affine registration and has not been extended to deformable registration tasks. Furthermore, this study was conducted using data acquired from a single institute with consistent imaging parameters. Because MRI contrast is highly dependent on scanner settings such as TR and TE, the generalizability of MsDCSwinT to data acquired using different MRI sequences or substantially different scan parameters has not yet been evaluated. In practical applications, additional training or fine-tuning may be required when deploying the model across sites with different acquisition protocols. Future work will include multi-center validation to assess performance across varied scanning environments. In this study, the test Dataset 1 was selected from the training dataset using cross-validation, while Dataset 2 comprised independent subjects from other studies and experimental conditions. This design supports intra-center generalization, and future work will focus on inter-center validation to evaluate robustness across imaging platforms and acquisition protocols. We also did not include comparisons with manual registration due to its time-consuming nature and reliance on expert interpretation. Nevertheless, incorporating such comparisons in future work could provide valuable insights into the method's performance in preclinical imaging studies.

### 4.2 Functional connectivity analysis

Seed-to-seed functional connectivity (FC) analysis remains a cornerstone in neuroimaging research, offering an atlas-based, hypothesis-driven framework to probe whole-brain functional organization. By utilizing predefined anatomical regions of interest (ROIs), seed-based FC decomposes complex neuroimaging data into spatially meaningful units, allowing researchers to quantify interregional communication and network-level brain function (Lupinsky et al., 2025; Sourty et al., 2024a,b; Nasseef et al., 2021; Karatas et al., 2021; Nasseef et al., 2019; Boulos et al., 2019; Hamida et al., 2018; Charbogne et al., 2017; Nasseef, 2015). Moreover, this approach is particularly powerful for assessing the influence of preprocessing steps such as spatial normalization and registration on downstream connectivity outcomes. In the present study, we combined a high-resolution, down-sampled anatomical rat brain atlas with a seed-to-seed FC framework to systematically evaluate the impact of multiple automated registration methods including our proposed unsupervised model MsDCSwinT against established techniques such as ANTS and alternative deep learning models (C2FViT, C2FGALF, and ConvNet).

Our findings demonstrate that the proposed MsDCSwinT method exhibits strong concordance with the classical ANTS algorithm, widely regarded as a gold standard for non-linear registration, while offering significant improvements in computational efficiency. Scatter plot comparisons (Figures 6A, C) and box plot analyses (Figure 6B) reveal that although the overall distributions of correlation coefficients (CC) are similar across methods, subtle yet meaningful variations in CC values suggest method-specific influences on alignment precision. These differences become more evident in pairwise group comparisons at the subject level, as assessed by two-sample t-tests (*p* = 0.05, FDR-corrected, *n* = 8 per group) (Figure 7). Importantly, the presence of these small but statistically discernible variations highlights the necessity of employing multimodal evaluation strategies during preprocessing validation, as reliance on single similarity metrics may obscure critical nuances (Nasseef et al., 2021, 2019; Boulos et al., 2019)). By integrating both statistical and visualization-based analyses (Figures 6, 7), our study underscores the need to benchmark AI-driven registration pipelines not only in terms of algorithmic accuracy but also based on their downstream impact on functional connectivity outcomes. Collectively, these results establish MsDCSwinT as a robust, scalable, and reliable preprocessing tool for rodent fMRI research, with potential to enhance reproducibility and methodological rigor in preclinical neuroimaging workflows.

#### 4.2.1 Limitations

For simplicity and clarity, we have taken the advantage of using h-values from the pairwise *t*-tests (*p* = 0.05, two-tailed, FDR-corrected, *n* = 8 per group) to highlight statistically significant differences in functional connectivity (Figure 7). Importantly, our primary aim was to identify the presence or absence of significant differences, rather than to assess the magnitude or strength of those differences in the form of p-values or tstat-values, which are more commonly interpreted in the context of biological or functional meaning (Lupinsky et al., 2025; Nasseef et al., 2021; Karatas et al., 2021; Nasseef et al., 2019; Boulos et al., 2019; Hamida et al., 2018; Charbogne et al., 2017). In this context, the binary nature of h-values (1 = significant vs. 0 = not significant) effectively serves our objective of comparing the different registration methods from a methodological perspective. Therefore, we deliberately chose not to present the associated p-values, as our interest lies in the existence of statistically significant differences, not in their relative quantification (Lupinsky et al., 2025; Nasseef et al., 2021, 2019; Boulos et al., 2019) or degree (Wang et al., 2025; Fuini et al., 2025) or effect size (Nasseef et al., 2018) or dynamic functional connectivity (Sourty et al., 2024a,b). Additionally, we did not perform further analysis such as hypothesis free independent component analysis (ICA) (Lupinsky et al., 2025; Nasseef et al., 2021; Karatas et al., 2021; Nasseef et al., 2019; Hamida et al., 2018; Charbogne et al., 2017) or directed (model based/model free) functional connectivity (Hamida et al., 2018; Charbogne et al., 2017) as these methods fall outside the primary scope of our current investigation. Given that our objective was to find the benchmark automated registration methods using atlas-based seed-to-seed correlation analysis, additional exploratory or directional techniques were deemed unnecessary. Equally, these alternative approaches are unlikely to provide further insights relevant to our methodological comparison and may instead introduce confounding variability unrelated to the core aim of evaluating registration performance.

## 5 Conclusion and future work

In this work, we presented a novel fMRI processing pipeline that combines a GAN-based denoising approach, transformer-based skull stripping, and affine registration methods. The proposed pipeline significantly enhances fMRI data preprocessing, with a main focus on registration in this study. It demonstrates superior performance in both registration accuracy and computational efficiency compared to traditional and learning-based methods. By integrating advanced deep learning models, such as GANs and transformers, the pipeline enables robust and automated processing, even in the absence of labelled data. In this paper, the primary emphasis was on the registration component. In future work, we plan to extend our evaluation by comparing the overall pipeline against other established fMRI preprocessing pipelines to further validate its effectiveness. Moreover, while we currently employ AFNI motion correction, we aim to develop our own motion correction method tailored to our specific pipeline. Motion artifacts are a common challenge in fMRI data, and we aim to develop strategies for reducing these artifacts to further improve the quality of processed fMRI data. Additionally, we plan to extend our work to incorporate deformable registration, allowing for more flexible alignment between images with complex transformations. Furthermore, we intend to test and optimize our pipeline on a wider variety of fMRI datasets, which will help evaluate its generalizability and applicability across different populations and experimental conditions. These extensions will enable our pipeline to become a more powerful and versatile tool for fMRI analysis, with potential applications in preclinical and research settings.

## Data Availability

The original contributions presented in the study are included in the article/supplementary material, further inquiries can be directed to the corresponding author.
